# Supplementary material for: Enhancement of Astaxanthin Biosynthesis in Oleaginous Yeast Yarrowia lipolytica via Microalgal Pathway
Source: Microorganisms. 2019 Oct 19;7(10):472. doi: 10.3390/microorganisms7100472 (PMC6843682; doi:10.3390/microorganisms7100472)
Supplement: Supplementary file 1 [file microorganisms-07-00472-s001.pdf]

Article

Enhancement of Astaxanthin Biosynthesis in Oleaginous Yeast *Yarrowia lipolytica* via Microalgal Pathway

Larissa Ribeiro Ramos Tramontin [1], Kanchana Rueksomtawin Kildegaard [1], Suresh Sudarsan [1] and Irina Borodina *

[1] The Novo Nordisk Foundation Center for Biosustainability, Technical University of Denmark, Kemitorvet 220, 2800 Kgs. Lyngby, Denmark; lartra@biosustain.dtu.dk (L.R.R.T.); kanchana@biophero.com (K.R.K.); sursud@biosustain.dtu.dk (S.S.)

* Correspondence: irbo@biosustain.dtu.dk

Supplementary figures

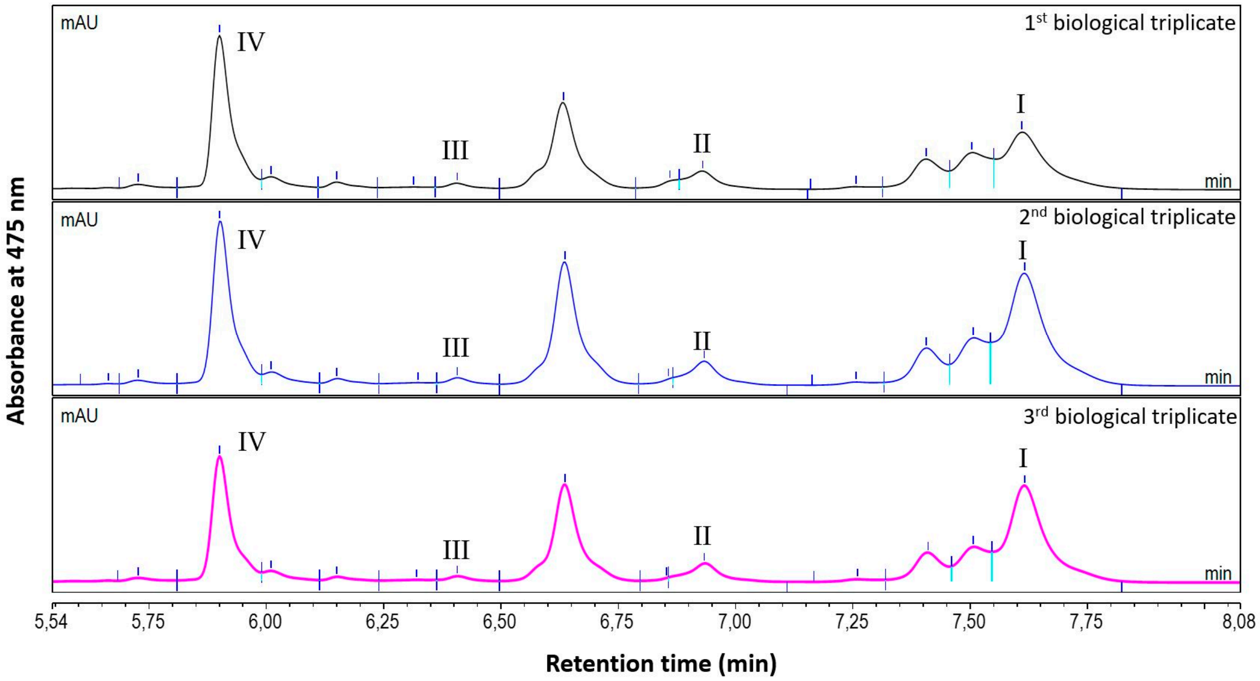

Figure S1. HPLC profile of carotenoids produced after 168h of fed-batch fermentation of ST7976 (iso 3). I: β-carotene; II: echinenone; III: canthaxanthin; IV: astaxanthin.

Supplementary tables

Table S1. Production of carotenoids by the strains generated in this study. Titers were quantified by HPLC analysis. Values are the average of the triplicates. The molar ratio (β-ketolase: β-hydroxylase) is indicated between brackets. The isolate number is indicated after the abbreviation 'iso'.

**Genotype**	**Strain ID**	**ß-carotene (mg/L)**	**±**	**Echinenone (mg/L)**	**±**	**Cantaxanthin (mg/L)**	**±**	**Astaxanthin (mg/L)**	**±**	**Dry cell (mg)**	**±**
Wild type	ST3683	0	0	0	0	0	0	0	0	13.93	1.38
XdCrtI ↑ - XdCrtYB ↑ XdCrtE ↑ - HMG1 ↑ PrSQS_50bp:SQS1	ST6899	88.85	11	0	0	0	0	0	0	21.00	1.35
XdCrtI ↑ - XdCrtYB ↑ XdCrtE ↑ - HMG1 ↑ PrSQS_50bp:SQS1 XdCrtE ↑	ST7433	132.02	11.28	0	0	0	0	0	0	18.13	1.16
XdCrtI ↑ - XdCrtYB ↑ XdCrtE ↑ - HMG1 ↑ PrSQS_50bp:SQS1 GGPPS7 ↑	ST7434	330.73	66.48	0	0	0	0	0	0	14.67	2.69
XdCrtI ↑ - XdCrtYB ↑ XdCrtE ↑ - HMG1 ↑ PrSQS_50bp:SQS1 GGPPS7 ↑ - CrtW ↑ ↑ ↑	ST7906	30.47	1.48	109.37	4.97	13.83	0.86	0	0	18.37	0.260
XdCrtI ↑ - XdCrtYB ↑ XdCrtE ↑ - HMG1 ↑ PrSQS_50bp:SQS1 GGPPS7 ↑ - HpBKT ↑ ↑ ↑	ST7972	82.73	14.15	55.87	3.53	30.85	2.65	0	0	18.47	0.291
XdCrtI ↑ - XdCrtYB ↑ XdCrtE ↑ - HMG1 ↑ PrSQS_50bp:SQS1 GGPPS7 ↑ - CrtW ↑ ↑ ↑ - PaCrtZ ↑ ↑ ↑	ST7927 (1:3) iso 6	174.22	7.50	13.02	0.21	4.67	0.12	17.56	0.19	16.20	0.058
XdCrtI ↑ - XdCrtYB ↑ XdCrtE ↑ - HMG1 ↑ - PSQS1_50bp:PsCrtW ↑ - PaCrtZ ↑ PrTefintron- PaCrtZ ↑ ↑ ↑	ST7400	45.08	2.31	27.58	3.11	2.21	0.29	15.80	0.83	13.83	1.82
XdCrtI ↑ - XdCrtYB ↑ XdCrtE ↑ - HMG1 ↑ PSQS1_50bp:SQS1 PsCrtW ↑ -PaCrtZ ↑	ST7403	22.66	2.80	22.17	5.20	1.70	0.41	18.10	0.64	13.87	0.28
XdCrtI ↑ - XdCrtYB ↑ XdCrtE ↑ - HMG1 ↑ PrSQS_50bp:SQS1 GGPPS7 ↑ - HpBKT ↑ ↑ ↑ -PaCrtZ ↑	ST7973 iso 4	25.22	1.41	42.79	1.92	9.28	1.01	10.24	0.10	16.37	0.29
XdCrtI ↑ - XdCrtYB ↑ XdCrtE ↑ - HMG1 ↑ PrSQS_50bp:SQS1 GGPPS7 ↑ - HpBKT ↑ ↑ ↑ -PaCrtZ ↑	ST7973 iso 5	51.96	11.72	38.91	3.80	13.60	3.11	9.38	0.62	16.70	0.15
XdCrtI ↑ - XdCrtYB ↑ XdCrtE ↑ - HMG1 ↑ PrSQS_50bp:SQS1 GGPPS7 ↑ - HpBKT ↑ ↑ ↑ -PaCrtZ ↑	ST7973 iso 6	67.87	7.29	29.66	3.07	15.95	1.47	9.80	0.53	16.10	0.23
XdCrtI ↑ - XdCrtYB ↑ XdCrtE ↑ - HMG1 ↑ PrSQS_50bp:SQS1 GGPPS7 ↑ - HpBKT ↑ ↑ ↑ -PaCrtZ ↑	ST7973 iso 12	75.01	11.55	35.05	1.47	15.61	2.11	10.77	0.20	16.17	0.12
XdCrtI ↑ - XdCrtYB ↑ XdCrtE ↑ - HMG1 ↑ PrSQS_50bp:SQS1 GGPPS7 ↑ - HpBKT ↑ ↑ ↑ -PaCrtZ ↑	ST7973 iso 16	46.69	3.28	71.56	3.97	18.70	1.37	8.37	0.31	16.07	0.27
XdCrtI ↑ - XdCrtYB ↑ XdCrtE ↑ - HMG1 ↑ PrSQS_50bp:SQS1 GGPPS7 ↑ - HpBKT ↑ ↑ ↑ -PaCrtZ ↑	ST7973 iso 28	24.58	4.37	51.15	0.89	15.67	0.40	5.23	0.42	15.90	0.45
XdCrtI ↑ - XdCrtYB ↑ XdCrtE ↑ - HMG1 ↑ PrSQS_50bp:SQS1 GGPPS7 ↑ - HpBKT ↑ ↑ ↑ -PaCrtZ ↑	ST7973 iso 29	17.22	2.02	62.45	5.71	18.55	2.70	4.75	0.21	16.97	0.93
XdCrtI ↑ - XdCrtYB ↑ XdCrtE ↑ - HMG1 ↑ PrSQS_50bp:SQS1 GGPPS7 ↑ - HpBKT ↑ ↑ ↑ -HpCrtZ ↑	ST7974 iso 1	44.28	2.24	53.94	2.43	6.48	0.57	16.23	0.32	20.97	3.18
XdCrtI ↑ - XdCrtYB ↑ XdCrtE ↑ - HMG1 ↑ PrSQS_50bp:SQS1 GGPPS7 ↑ - HpBKT ↑ ↑ ↑ -HpCrtZ ↑	ST7974 iso 7	91.92	2.77	49.49	1.02	9.12	0.49	10.85	0.08	17.03	0.03

XdCrtI↑ - XdCrtYB↑ XdCrtE↑ - HMG1↑ PrSQS_50bp:SQS1 GGPPS7↑ - HpBKT↑↑↑ -HpCrtZ↑	ST7974 iso 11	39.99	2.31	47.58	3.54	3.43	0.53	20.41	0.83	17.67	0.19
XdCrtI↑ - XdCrtYB↑ XdCrtE↑ - HMG1↑ PrSQS_50bp:SQS1 GGPPS7↑ - HpBKT↑↑↑ -HpCrtZ↑	ST7974 iso 14	30.77	1.98	41.28	2.07	2.19	0.32	19.62	0.27	17.67	0.27
XdCrtI↑ - XdCrtYB↑ XdCrtE↑ - HMG1↑ PrSQS_50bp:SQS1 GGPPS7↑ - HpBKT↑↑↑ -HpCrtZ↑	ST7974 iso 16	31.88	1.26	39.50	1.73	1.81	0.32	18.50	0.40	17.53	0.62
XdCrtI↑ - XdCrtYB↑ XdCrtE↑ - HMG1↑ PrSQS_50bp:SQS1 GGPPS7↑ - HpBKT↑↑↑ -HpCrtZ↑	ST7974 iso 20	50.22	3.61	44.12	2.36	8.11	0.72	16.29	0.24	17.77	0.47
XdCrtI↑ - XdCrtYB↑ XdCrtE↑ - HMG1↑ PrSQS_50bp:SQS1 GGPPS7↑ - HpBKT↑↑↑ -PaCrtZ↑↑↑	ST7975 (1:1) iso 3	28.63	1.72	48.50	2.50	20.94	0.88	3.77	0.12	16.97	0.53
XdCrtI↑ - XdCrtYB↑ XdCrtE↑ - HMG1↑ PrSQS_50bp:SQS1 GGPPS7↑ - HpBKT↑↑↑ -PaCrtZ↑↑↑	ST7975 (1:1) iso 4	39.72	7.80	48.09	2.07	14.91	1.13	9.51	0.09	16.83	0.23
XdCrtI↑ - XdCrtYB↑ XdCrtE↑ - HMG1↑ PrSQS_50bp:SQS1 GGPPS7↑ - HpBKT↑↑↑ -PaCrtZ↑↑↑	ST7975 (1:1) iso 5	16.47	1.19	51.58	2.87	25.21	1.21	1.05	0.01	17.57	1.25
XdCrtI↑ - XdCrtYB↑ XdCrtE↑ - HMG1↑ PrSQS_50bp:SQS1 GGPPS7↑ - HpBKT↑↑↑ -PaCrtZ↑↑↑	ST7975 (1:1) iso 6	14.77	1.16	54.91	1.87	33.50	1.13	0.00	0.00	18.07	2.02
XdCrtI↑ - XdCrtYB↑ XdCrtE↑ - HMG1↑ PrSQS_50bp:SQS1 GGPPS7↑ - HpBKT↑↑↑ -PaCrtZ↑↑↑	ST7975 (1:1) iso 7	27.79	2.73	50.21	1.33	23.81	1.15	2.09	0.18	16.17	0.32
XdCrtI↑ - XdCrtYB↑ XdCrtE↑ - HMG1↑ PrSQS_50bp:SQS1 GGPPS7↑ - HpBKT↑↑↑ -PaCrtZ↑↑↑	ST7975 (1:1) iso 8	32.13	1.60	56.51	3.30	20.00	1.56	4.56	0.14	15.73	0.44
XdCrtI↑ - XdCrtYB↑ XdCrtE↑ - HMG1↑ PrSQS_50bp:SQS1 GGPPS7↑ - HpBKT↑↑↑ -PaCrtZ↑↑↑	ST7975 (1:1) iso 9	10.55	0.24	63.34	1.56	33.34	1.05	1.21	0.12	16.50	0.60
XdCrtI↑ - XdCrtYB↑ XdCrtE↑ - HMG1↑ PrSQS_50bp:SQS1 GGPPS7↑ - HpBKT↑↑↑ -PaCrtZ↑↑↑	ST7975 (1:2) iso 1	177.09	15.37	135.54	8.36	88.62	3.39	0.00	0.00	16.70	0.60
XdCrtI↑ - XdCrtYB↑ XdCrtE↑ - HMG1↑ PrSQS_50bp:SQS1 GGPPS7↑ - HpBKT↑↑↑ -PaCrtZ↑↑↑	ST7975 (1:2) iso 3	19.88	2.01	55.16	2.41	32.01	2.19	0.00	0.00	15.67	0.07
XdCrtI↑ - XdCrtYB↑ XdCrtE↑ - HMG1↑ PrSQS_50bp:SQS1 GGPPS7↑ - HpBKT↑↑↑ -PaCrtZ↑↑↑	ST7975 (1:2) iso 6	32.39	4.16	48.00	3.12	11.98	1.56	11.02	0.11	15.70	0.31
XdCrtI ↑ - XdCrtYB ↑ XdCrtE ↑ - HMG1 ↑- PrSQS_50bp:SQS1 GGPPS7 ↑ - HpBKT ↑ ↑ ↑ -PaCrtZ ↑ ↑ ↑	ST7975 (1:2) iso 7	17.84	1.53	63.11	3.23	20.53	1.05	0.00	0.00	15.93	0.15
XdCrtI ↑ - XdCrtYB ↑ XdCrtE ↑ - HMG1 ↑- PrSQS_50bp:SQS1 GGPPS7 ↑ - HpBKT ↑ ↑ ↑ -PaCrtZ ↑ ↑ ↑	ST7975 (1:2) iso 11	231.41	25.97	43.72	3.27	18.90	1.39	0.00	0.00	14.70	0.17
XdCrtI ↑ - XdCrtYB ↑ XdCrtE ↑ - HMG1 ↑- PrSQS_50bp:SQS1 GGPPS7 ↑ - HpBKT ↑ ↑ ↑ -PaCrtZ ↑ ↑ ↑	ST7975 (1:2) iso 12	141.87	73.55	32.19	3.57	15.89	1.33	12.86	0.31	15.90	0.49
XdCrtI ↑ - XdCrtYB ↑ XdCrtE ↑ - HMG1 ↑- PrSQS_50bp:SQS1 GGPPS7 ↑ - HpBKT ↑ ↑ ↑ -PaCrtZ ↑ ↑ ↑	ST7975 (1:2) iso 14	18.52	3.65	64.70	3.65	28.81	1.48	0.00	0.00	15.93	0.32
XdCrtI ↑ - XdCrtYB ↑ XdCrtE ↑ - HMG1 ↑- PrSQS_50bp:SQS1 GGPPS7 ↑ - HpBKT ↑ ↑ ↑ -PaCrtZ ↑ ↑ ↑	ST7975 (1:3) iso 1	33.27	1.55	57.80	4.83	29.79	2.43	8.97	0.64	17.23	0.03
XdCrtI ↑ - XdCrtYB ↑ XdCrtE ↑ - HMG1 ↑- PrSQS_50bp:SQS1 GGPPS7 ↑ - HpBKT ↑ ↑ ↑ -PaCrtZ ↑ ↑ ↑	ST7975 (1:3) iso 2	201.69	54.85	55.25	3.16	25.83	1.33	1.88	0.24	17.10	0.65
XdCrtI ↑ - XdCrtYB ↑ XdCrtE ↑ - HMG1 ↑- PrSQS_50bp:SQS1 GGPPS7 ↑ - HpBKT ↑ ↑ ↑ -PaCrtZ ↑ ↑ ↑	ST7975 (1:3) iso 3	12.70	2.12	81.20	10.36	45.24	6.72	0.38	0.38	17.20	0.25
XdCrtI ↑ - XdCrtYB ↑ XdCrtE ↑ - HMG1 ↑- PrSQS_50bp:SQS1 GGPPS7 ↑ - HpBKT ↑ ↑ ↑ -PaCrtZ ↑ ↑ ↑	ST7975 (1:3) iso 5	14.75	1.08	105.79	8.48	52.00	4.18	2.12	0.07	17.07	0.22
XdCrtI ↑ - XdCrtYB ↑ XdCrtE ↑ - HMG1 ↑- PrSQS_50bp:SQS1 GGPPS7 ↑ - HpBKT ↑ ↑ ↑ -PaCrtZ ↑ ↑ ↑	ST7975 (1:3) iso 6	23.31	2.56	113.38	10.32	35.18	1.93	0.33	0.33	15.07	0.22
XdCrtI ↑ - XdCrtYB ↑ XdCrtE ↑ - HMG1 ↑- PrSQS_50bp:SQS1 GGPPS7 ↑ - HpBKT ↑ ↑ ↑ -PaCrtZ ↑ ↑ ↑	ST7975 (1:3) iso 11	4.62	0.68	135.25	7.14	39.31	1.72	1.65	0.19	15.73	0.88

XdCrtI↑- *XdCrtYB*↑ *XdCrtE*↑- *HMG1*↑- *PrSQS_50bp:SQS1 GGPPS7*↑- *HpBKT*↑↑↑-*PaCrtZ*↑↑↑	ST7975 (1:3) iso 15	29.30	0.82	35.81	12.80	22.44	1.36	13.48	0.39	16.37	0.29
XdCrtI↑- *XdCrtYB*↑ *XdCrtE*↑- *HMG1*↑- *PrSQS_50bp:SQS1 GGPPS7*↑- *HpBKT*↑↑↑-*HpCrtZ*↑↑↑	ST7976 (1:1) iso 3	163.32	12.75	32.32	5.73	0.89	0.51	44.34	1.40	14.80	0.46
XdCrtI↑- *XdCrtYB*↑ *XdCrtE*↑- *HMG1*↑- *PrSQS_50bp:SQS1 GGPPS7*↑- *HpBKT*↑↑↑-*HpCrtZ*↑↑↑	ST7976 (1:1) iso 9	221.91	25.67	28.62	4.96	7.14	1.46	29.63	1.40	16.07	0.20
XdCrtI↑- *XdCrtYB*↑ *XdCrtE*↑- *HMG1*↑- *PrSQS_50bp:SQS1 GGPPS7*↑- *HpBKT*↑↑↑-*HpCrtZ*↑↑↑	ST7976 (1:1) iso 11	186.55	7.79	26.65	4.47	7.30	1.41	26.04	1.64	14.90	0.31
XdCrtI↑- *XdCrtYB*↑ *XdCrtE*↑- *HMG1*↑- *PrSQS_50bp:SQS1 GGPPS7*↑- *HpBKT*↑↑↑-*HpCrtZ*↑↑↑	ST7976 (1:1) iso 12	135.50	26.42	28.42	5.76	7.40	1.81	27.86	1.34	15.97	0.61
XdCrtI↑- *XdCrtYB*↑ *XdCrtE*↑- *HMG1*↑- *PrSQS_50bp:SQS1 GGPPS7*↑- *HpBKT*↑↑↑-*HpCrtZ*↑↑↑	ST7976 (1:1) iso 13	146.95	29.73	29.80	2.13	7.10	1.22	26.94	0.25	15.83	0.18
XdCrtI↑- *XdCrtYB*↑ *XdCrtE*↑- *HMG1*↑- *PrSQS_50bp:SQS1 GGPPS7*↑- *HpBKT*↑↑↑-*HpCrtZ*↑↑↑	ST7976 (1:1) iso 14	23.69	1.88	79.04	11.96	35.10	0.87	12.28	0.93	16.13	0.58
XdCrtI↑- *XdCrtYB*↑ *XdCrtE*↑- *HMG1*↑- *PrSQS_50bp:SQS1 GGPPS7*↑- *HpBKT*↑↑↑-*HpCrtZ*↑↑↑	ST7976 (1:1) iso 20	65.13	2.50	19.89	1.12	3.27	1.68	27.60	2.83	18.03	0.54
XdCrtI↑- *XdCrtYB*↑ *XdCrtE*↑- *HMG1*↑- *PrSQS_50bp:SQS1 GGPPS7*↑- *HpBKT*↑↑↑-*HpCrtZ*↑↑↑	ST7976 (1:2) iso 3	188.16	28.54	94.39	9.77	48.11	1.79	0.00	0.00	15.03	1.01
XdCrtI↑- *XdCrtYB*↑ *XdCrtE*↑- *HMG1*↑- *PrSQS_50bp:SQS1 GGPPS7*↑- *HpBKT*↑↑↑-*HpCrtZ*↑↑↑	ST7976 (1:2) iso 11	225.80	33.68	95.30	1.79	42.12	1.88	0.43	0.43	17.23	0.62
XdCrtI↑- *XdCrtYB*↑ *XdCrtE*↑- *HMG1*↑- *PrSQS_50bp:SQS1 GGPPS7*↑- *HpBKT*↑↑↑-*HpCrtZ*↑↑↑	ST7976 (1:2) iso 12	96.81	23.34	50.48	8.62	4.20	0.91	41.37	1.35	17.00	0.20
XdCrtI↑- *XdCrtYB*↑ *XdCrtE*↑- *HMG1*↑- *PrSQS_50bp:SQS1 GGPPS7*↑- *HpBKT*↑↑↑-*HpCrtZ*↑↑↑	ST7976 (1:2) iso 13	24.87	1.79	65.38	2.89	37.65	0.80	0.00	0.00	16.60	0.31
XdCrtI↑- *XdCrtYB*↑ *XdCrtE*↑- *HMG1*↑- *PrSQS_50bp:SQS1 GGPPS7*↑- *HpBKT*↑↑↑-*HpCrtZ*↑↑↑	ST7976 (1:2) iso 14	117.23	35.00	51.42	3.46	16.31	1.12	23.45	1.53	16.50	0.76
XdCrtI↑- *XdCrtYB*↑ *XdCrtE*↑- *HMG1*↑- *PrSQS_50bp:SQS1 GGPPS7*↑- *HpBKT*↑↑↑-*HpCrtZ*↑↑↑	ST7976 (1:2) iso 15	137.55	19.51	72.54	10.99	49.55	2.42	0.00	0.00	17.20	0.36
XdCrtI↑- *XdCrtYB*↑ *XdCrtE*↑- *HMG1*↑- *PrSQS_50bp:SQS1 GGPPS7*↑- *HpBKT*↑↑↑-*HpCrtZ*↑↑↑	ST7976 (1:2) iso 16	238.94	7.65	47.46	2.94	2.61	0.32	42.92	0.25	17.43	0.30
XdCrtI↑- *XdCrtYB*↑ *XdCrtE*↑- *HMG1*↑- *PrSQS_50bp:SQS1 GGPPS7*↑- *HpBKT*↑↑↑-*HpCrtZ*↑↑↑	ST7976 (1:3) iso 3	269.62	45.75	96.81	13.00	51.04	4.42	4.61	1.99	17.33	0.92
XdCrtI↑- *XdCrtYB*↑ *XdCrtE*↑- *HMG1*↑- *PrSQS_50bp:SQS1 GGPPS7*↑- *HpBKT*↑↑↑-*HpCrtZ*↑↑↑	ST7976 (1:3) iso 10	358.35	25.18	61.70	22.75	14.93	1.22	25.28	3.07	15.77	0.22
XdCrtI↑- *XdCrtYB*↑ *XdCrtE*↑- *HMG1*↑- *PrSQS_50bp:SQS1 GGPPS7*↑- *HpBKT*↑↑↑-*HpCrtZ*↑↑↑	ST7976 (1:3) iso 11	206.45	46.96	147.01	14.31	35.63	1.17	13.00	1.00	15.67	0.38
XdCrtI↑- *XdCrtYB*↑ *XdCrtE*↑- *HMG1*↑- *PrSQS_50bp:SQS1 GGPPS7*↑- *HpBKT*↑↑↑-*HpCrtZ*↑↑↑	ST7976 (1:3) iso 12	225.81	17.94	60.42	0.64	60.17	2.78	3.21	0.27	14.80	0.15
XdCrtI↑- *XdCrtYB*↑ *XdCrtE*↑- *HMG1*↑- *PrSQS_50bp:SQS1 GGPPS7*↑- *HpBKT*↑↑↑-*HpCrtZ*↑↑↑	ST7976 (1:3) iso 15	253.72	5.10	19.00	2.85	27.91	0.75	18.01	0.78	19.00	0.40
XdCrtI↑- *XdCrtYB*↑ *XdCrtE*↑- *HMG1*↑- *PrSQS_50bp:SQS1 GGPPS7*↑- *HpBKT*↑↑↑-*HpCrtZ*↑↑↑	ST7976 (1:3) iso 17	400.36	15.44	96.09	32.36	35.65	0.67	12.84	1.17	16.10	0.49
XdCrtI↑- *XdCrtYB*↑ *XdCrtE*↑- *HMG1*↑- *PrSQS_50bp:SQS1 GGPPS7*↑- *HpBKT*↑↑↑-*HpCrtZ*↑↑↑	ST7976 (1:3) iso 18	165.23	23.04	11.86	7.93	15.36	10.32	10.27	0.91	19.77	0.19

XdCrtI ↑ - XdCrtYB ↑ XdCrtE ↑ - HMG1 ↑- PrSQS_50bp:SQS1 GGPPS7 ↑ - PsCrtW ↑ ↑ ↑-HpCrtZ ↑	ST7926 iso 4	38.53	3.13	175.83	15.85	10.88	3.20	7.56	1.49	15.43	0.83
XdCrtI ↑ - XdCrtYB ↑ XdCrtE ↑ - HMG1 ↑- PrSQS_50bp:SQS1 GGPPS7 ↑ - PsCrtW ↑ ↑ ↑-HpCrtZ ↑	ST7926 iso 5	47.81	3.86	192.87	2.90	7.76	0.51	12.71	0.16	19.70	0.25
XdCrtI ↑ - XdCrtYB ↑ XdCrtE ↑ - HMG1 ↑- PrSQS_50bp:SQS1 GGPPS7 ↑ - PsCrtW ↑ ↑ ↑-HpCrtZ ↑	ST7926 iso 8	204.15	62.72	123.55	2.91	11.42	0.36	1.95	0.10	17.97	0.15
XdCrtI ↑ - XdCrtYB ↑ XdCrtE ↑ - HMG1 ↑- PrSQS_50bp:SQS1 GGPPS7 ↑ - PsCrtW ↑ ↑ ↑-HpCrtZ ↑	ST7926 iso 9	1038.46	47.05	0.14	0.01	0.00	0.00	0.00	0.00	21.23	0.03
XdCrtI ↑ - XdCrtYB ↑ XdCrtE ↑ - HMG1 ↑- PrSQS_50bp:SQS1 GGPPS7 ↑ - PsCrtW ↑ ↑ ↑-HpCrtZ ↑	ST7926 iso 10	31.35	2.35	199.24	7.56	14.73	1.77	10.40	1.69	15.73	0.27
XdCrtI ↑ - XdCrtYB ↑ XdCrtE ↑ - HMG1 ↑- PrSQS_50bp:SQS1 GGPPS7 ↑ - PsCrtW ↑ ↑ ↑-HpCrtZ ↑	ST7926 iso 12	146.57	5.49	78.15	13.97	11.80	0.33	3.03	0.19	17.00	0.23
XdCrtI ↑ - XdCrtYB ↑ XdCrtE ↑ - HMG1 ↑- PrSQS_50bp:SQS1 GGPPS7 ↑ - PsCrtW ↑ ↑ ↑-PaCrtZ ↑	ST7925 iso 1	3.07	0.09	55.36	0.30	15.61	0.29	0.00	0.00	14.57	0.17
XdCrtI ↑ - XdCrtYB ↑ XdCrtE ↑ - HMG1 ↑- PrSQS_50bp:SQS1 GGPPS7 ↑ - PsCrtW ↑ ↑ ↑-PaCrtZ ↑	ST7925 iso 2	3.66	0.58	39.99	6.66	0.61	0.61	10.84	1.50	13.40	0.53
XdCrtI ↑ - XdCrtYB ↑ XdCrtE ↑ - HMG1 ↑- PrSQS_50bp:SQS1 GGPPS7 ↑ - PsCrtW ↑ ↑ ↑-PaCrtZ ↑ ↑ ↑	ST7627 (1:1) iso 1	4.50	1.55	156.90	23.50	12.25	3.31	11.90	0.66	17.07	0.29
XdCrtI ↑ - XdCrtYB ↑ XdCrtE ↑ - HMG1 ↑- PrSQS_50bp:SQS1 GGPPS7 ↑ - PsCrtW ↑ ↑ ↑- PaCrtZ ↑ ↑ ↑	ST7627 (1:1) iso 2	5.60	1.03	99.09	2.20	4.74	2.37	16.74	0.33	16.63	0.22
XdCrtI ↑ - XdCrtYB ↑ XdCrtE ↑ - HMG1 ↑- PrSQS_50bp:SQS1 GGPPS7 ↑ - PsCrtW ↑ ↑ ↑- PaCrtZ ↑ ↑ ↑	ST7627 (1:1) iso 4	8.47	3.35	147.93	4.94	21.53	1.29	7.46	1.20	13.87	0.22
XdCrtI ↑ - XdCrtYB ↑ XdCrtE ↑ - HMG1 ↑- PrSQS_50bp:SQS1 GGPPS7 ↑ - PsCrtW ↑ ↑ ↑- PaCrtZ ↑ ↑ ↑	ST7627 (1:1) iso 7	53.77	11.38	64.60	3.27	4.91	0.47	12.49	0.50	16.87	0.32
XdCrtI ↑ - XdCrtYB ↑ XdCrtE ↑ - HMG1 ↑- PrSQS_50bp:SQS1 GGPPS7 ↑ - PsCrtW ↑ ↑ ↑- PaCrtZ ↑ ↑ ↑	ST7627 (1:2) iso 2	2.78	0.91	143.08	16.37	8.50	1.84	12.93	0.56	15.77	0.84
XdCrtI ↑ - XdCrtYB ↑ XdCrtE ↑ - HMG1 ↑- PrSQS_50bp:SQS1 GGPPS7 ↑ - PsCrtW ↑ ↑ ↑- PaCrtZ ↑ ↑ ↑	ST7627 (1:2) iso 3	3.81	0.37	14.65	1.64	0.56	0.30	4.69	0.78	15.97	1.47
XdCrtI ↑ - XdCrtYB ↑ XdCrtE ↑ - HMG1 ↑- PrSQS_50bp:SQS1 GGPPS7 ↑ - PsCrtW ↑ ↑ ↑- PaCrtZ ↑ ↑ ↑	ST7627 (1:2) iso 4	11.91	1.54	72.51	12.17	5.76	2.10	17.28	0.92	14.43	0.95
XdCrtI ↑ - XdCrtYB ↑ XdCrtE ↑ - HMG1 ↑- PrSQS_50bp:SQS1 GGPPS7 ↑ - PsCrtW ↑ ↑ ↑- PaCrtZ ↑ ↑ ↑	ST7627 (1:2) iso 5	25.46	1.49	64.55	4.95	3.19	0.45	18.81	1.30	15.33	0.78
XdCrtI ↑ - XdCrtYB ↑ XdCrtE ↑ - HMG1 ↑- PrSQS_50bp:SQS1 GGPPS7 ↑ - PsCrtW ↑ ↑ ↑- PaCrtZ ↑ ↑ ↑	ST7627 (1:3) iso 1	30.71	6.04	98.48	18.05	8.82	2.63	15.23	0.97	16.03	0.93
XdCrtI ↑ - XdCrtYB ↑ XdCrtE ↑ - HMG1 ↑- PrSQS_50bp:SQS1 GGPPS7 ↑ - PsCrtW ↑ ↑ ↑- PaCrtZ ↑ ↑ ↑	ST7627 (1:3) iso 2	146.12	20.67	0.00	0.00	0.47	0.23	32.86	1.20	15.13	0.95
XdCrtI ↑ - XdCrtYB ↑ XdCrtE ↑ - HMG1 ↑- PrSQS_50bp:SQS1 GGPPS7 ↑ - PsCrtW ↑ ↑ ↑- PaCrtZ ↑ ↑ ↑	ST7627 (1:3) iso 5	303.73	15.75	10.43	0.73	0.77	0.07	8.13	0.30	14.43	0.84
XdCrtI ↑ - XdCrtYB ↑ XdCrtE ↑ - HMG1 ↑- PrSQS_50bp:SQS1 GGPPS7 ↑ - PsCrtW ↑ ↑ ↑- PaCrtZ ↑ ↑ ↑	ST7627 (1:3) iso 6	136.57	37.55	0.00	0.00	0.48	0.24	44.98	4.72	14.40	1.05
XdCrtI ↑ - XdCrtYB ↑ XdCrtE ↑ - HMG1 ↑- PrSQS_50bp:SQS1 GGPPS7 ↑ - PsCrtW ↑ ↑ ↑- HpCrtZ ↑ ↑ ↑	ST7928 (1:1) iso 6	13.27	1.62	134.15	16.49	2.69	0.60	18.90	2.52	16.13	0.81
XdCrtI ↑ - XdCrtYB ↑ XdCrtE ↑ - HMG1 ↑- PrSQS_50bp:SQS1 GGPPS7 ↑ - PsCrtW ↑ ↑ ↑- HpCrtZ ↑ ↑ ↑	ST7928 (1:1) iso 10	7.14	1.62	107.96	28.06	3.37	0.99	13.55	4.78	14.80	1.93

XdCrtI ↑ - XdCrtYB ↑ XdCrtE ↑ - HMG1 ↑-<br>PrSQS_50bp:SQS1 GGPPS7 ↑ - PsCrtW ↑ ↑ ↑- HpCrtZ ↑ ↑ ↑	ST7928 (1:1)<br>iso 11	17.97	0.3<br>9	63.90	10.<br>95	0.20	0.1<br>0	19.90	4.<br>11	15.50	0.9<br>6
XdCrtI ↑ - XdCrtYB ↑ XdCrtE ↑ - HMG1 ↑-<br>PrSQS_50bp:SQS1 GGPPS7 ↑ - PsCrtW ↑ ↑ ↑- HpCrtZ ↑ ↑ ↑	ST7928 (1:1)<br>iso 15	18.63	0.6<br>2	50.15	7.4<br>2	1.19	0.1<br>8	17.91	3.<br>44	14.13	0.6<br>9
XdCrtI ↑ - XdCrtYB ↑ XdCrtE ↑ - HMG1 ↑-<br>PrSQS_50bp:SQS1 GGPPS7 ↑ - PsCrtW ↑ ↑ ↑- HpCrtZ ↑ ↑ ↑	ST7928 (1:2)<br>iso 1	19.74	1.9<br>2	136.16	4.0<br>4	7.48	1.4<br>8	17.39	0.<br>37	14.10	0.6<br>7
XdCrtI ↑ - XdCrtYB ↑ XdCrtE ↑ - HMG1 ↑-<br>PrSQS_50bp:SQS1 GGPPS7 ↑ - PsCrtW ↑ ↑ ↑- HpCrtZ ↑ ↑ ↑	ST7928 (1:2)<br>iso 4	4.39	0.1<br>3	62.06	14.<br>82	1.34	0.3<br>0	18.05	4.<br>21	15.20	1.0<br>6
XdCrtI ↑ - XdCrtYB ↑ XdCrtE ↑ - HMG1 ↑-<br>PrSQS_50bp:SQS1 GGPPS7 ↑ - PsCrtW ↑ ↑ ↑- HpCrtZ ↑ ↑ ↑	ST7928 (1:2)<br>iso 5	51.55	13.<br>13	53.42	11.<br>68	1.59	0.3<br>3	16.60	3.<br>31	14.00	1.7<br>6
XdCrtI ↑ - XdCrtYB ↑ XdCrtE ↑ - HMG1 ↑-<br>PrSQS_50bp:SQS1 GGPPS7 ↑ - PsCrtW ↑ ↑ ↑- HpCrtZ ↑ ↑ ↑	ST7928 (1:2)<br>iso 8	26.94	8.3<br>5	60.49	18.<br>83	0.17	0.1<br>0	18.77	4.<br>61	15.67	1.2<br>0
XdCrtI ↑ - XdCrtYB ↑ XdCrtE ↑ - HMG1 ↑-<br>PrSQS_50bp:SQS1 GGPPS7 ↑ - PsCrtW ↑ ↑ ↑- HpCrtZ ↑ ↑ ↑	ST7928 (1:3)<br>iso 2	14.57	2.6<br>9	26.06	5.0<br>5	0.07	0.0<br>7	15.90	3.<br>03	14.57	0.5<br>2
XdCrtI ↑ - XdCrtYB ↑ XdCrtE ↑ - HMG1 ↑-<br>PrSQS_50bp:SQS1 GGPPS7 ↑ - PsCrtW ↑ ↑ ↑- HpCrtZ ↑ ↑ ↑	ST7928 (1:3)<br>iso 9	30.24	2.6<br>1	22.17	1.6<br>4	0.00	0.0<br>0	14.36	0.<br>79	14.20	0.2<br>3
XdCrtI ↑ - XdCrtYB ↑ XdCrtE ↑ - HMG1 ↑-<br>PrSQS_50bp:SQS1 GGPPS7 ↑ - PsCrtW ↑ ↑ ↑- HpCrtZ ↑ ↑ ↑	ST7928 (1:3)<br>iso 10	10.60	1.9<br>6	61.02	10.<br>06	0.00	0.0<br>0	19.74	1.<br>74	15.73	0.5<br>2
XdCrtI ↑ - XdCrtYB ↑ XdCrtE ↑ - HMG1 ↑-<br>PrSQS_50bp:SQS1 GGPPS7 ↑ - PsCrtW ↑ ↑ ↑- HpCrtZ ↑ ↑ ↑	ST7928 (1:3)<br>iso 12	11.83	3.6<br>2	75.97	20.<br>06	0.00	0.0<br>0	23.48	4.<br>47	17.30	1.4<br>0

Table S2. List of *Yarrowia lipolytica* engineered strains. Single black arrow (↑) represents a single integration event; triple black arrow (↑ ↑ ↑) represents multiple integration events.

Strain ID	Genotype	Parent strain	Integrated Plasmid	Reference
ST6899	ST6899 (XdCrtI ↑ - XdCrtYB ↑ XdCrtE ↑ - HMG1 ↑ PrSQS_50bp:SQS1 loop out HphMX)			[23]
ST7400	XdCrtI ↑ - XdCrtYB ↑ XdCrtE ↑ - HMG1 ↑ - PrSQS1_50bp:PsCrtW ↑ -PaCrtZ ↑ - PrTefintron-PaCrtZ ↑ ↑ ↑			[23]
ST7403	XdCrtI ↑ - XdCrtYB ↑ XdCrtE ↑ - HMG1 ↑ - PrSQS1_50bp:SQS1 PsCrtW ↑ -PaCrtZ ↑			[23]
ST7433	ST7433 (XdCrtI ↑ - XdCrtYB ↑ XdCrtE ↑ - HMG1 ↑ PrSQS_50bp:SQS1 XdCrtE ↑)	ST6899	pCfB7646	This study
ST7434	ST7434 (XdCrtI ↑ - XdCrtYB ↑ XdCrtE ↑ - HMG1 ↑ PrSQS_50bp:SQS1 GGPPS7 ↑)	ST6899	pCfB7645	This study
ST7906	ST7906 (XdCrtI ↑ - XdCrtYB ↑ XdCrtE ↑ - HMG1 ↑ PrSQS_50bp:SQS1 GGPPS7 ↑ PsCrtW ↑ ↑ ↑)	ST7434	pCfB7063	This study
ST7925	ST7925 (XdCrtI ↑ - XdCrtYB ↑ XdCrtE ↑ - HMG1 ↑ PrSQS_50bp:SQS1 GGPPS7 ↑ PsCrtW ↑ ↑ ↑ PaCrtZ ↑)	ST7906	pCfB7815	This study
ST7926	ST7926 (XdCrtI ↑ - XdCrtYB ↑ XdCrtE ↑ - HMG1 ↑ PrSQS_50bp:SQS1 GGPPS7 ↑ PsCrtW ↑ ↑ ↑ HpCrtZ ↑)	ST7906	pCfB7883	This study
ST7927	ST7927 (XdCrtI ↑ - XdCrtYB ↑ XdCrtE ↑ - HMG1 ↑ PrSQS_50bp:SQS1 GGPPS7 ↑ PsCrtW ↑ ↑ ↑ PaCrtZ ↑ ↑ ↑)	ST7434	pCfB7063 and pCfB7064	This study
ST7928	ST7928 (XdCrtI ↑ - XdCrtYB ↑ XdCrtE ↑ - HMG1 ↑ PrSQS_50bp:SQS1 GGPPS7 ↑ PsCrtW ↑ ↑ ↑ HpCrtZ ↑ ↑ ↑)	ST7434	pCfB7063 and pCfB7884	This study
ST7972	ST7972 (XdCrtI ↑ - XdCrtYB ↑ XdCrtE ↑ - HMG1 ↑ PrSQS_50bp:SQS1 GGPPS7 ↑ HpBKT ↑ ↑ ↑)	ST7434	pCfB8027	This study
ST7973	ST7973 (XdCrtI ↑ - XdCrtYB ↑ XdCrtE ↑ - HMG1 ↑ PrSQS_50bp:SQS1 GGPPS7 ↑ HpBKT ↑ ↑ ↑ PaCrtZ ↑)	ST7972	pCfB7815	This study
ST7974	ST7974 (XdCrtI ↑ - XdCrtYB ↑ XdCrtE ↑ - HMG1 ↑ PrSQS_50bp:SQS1 GGPPS7 ↑ HpBKT ↑ ↑ ↑ HpCrtZ ↑)	ST7972	pCfB7883	This study
ST7975	ST7975 (XdCrtI ↑ - XdCrtYB ↑ XdCrtE ↑ - HMG1 ↑ PrSQS_50bp:SQS1 GGPPS7 ↑ HpBKT ↑ ↑ ↑ PaCrtZ ↑ ↑ ↑)	ST7434	pCfB8027 and pCfB7064	This study
ST7976	ST7976 (XdCrtI ↑ - XdCrtYB ↑ XdCrtE ↑ - HMG1 ↑ PrSQS_50bp:SQS1 GGPPS7 ↑ HpBKT ↑ ↑ ↑ HpCrtZ ↑ ↑ ↑)	ST7434	pCfB8027 and pCfB7884	This study

Table S3. List of plasmids used in this study. The plasmids were constructed by USER cloning, inserting BioBricks into parental plasmid.

ID	Description	Parental plasmid	Inserted BioBricks	Reference
pCfB4784	pIntE-2-NatMX-TPex20-TLip2			[39]
pCfB5219	pIntE-4-HphMX-TPex20-TLip2			[39]
pCfB5313	prDNA-Ura3d1-TPex20-TLip2			[23]
pCfB7063	prDNA-Ura3d1-PrTefintron->PsCrtW-TLip2	pCfB5313	BB02093, BB02220	[23]
pCfB7064	prDNA-Ura3d1-PrTefintron-PaCrtZ-TLip2	pCfB5313	BB02093, BB02221	[23]
pCfB7645	pIntF-2-NatMX-PrFBA1->SsGGPPS7-TLip2	pCfB4784	BB01559, BB02142	This study
pCfB7646	pIntF-2-NatMX-PrFBA1->XdCrtE-TLip2	pCfB4784	BB01559, BB01569	This study
pCfB7815	pIntE-4-HphMX-TPex20-PrTefintron->PaCrtZ-TLip2	pCfB5219	BB02093, BB02221	This study
pCfB7883	pIntE-4-HphMX-TPex20-PrTefintron->HpCrtZ-TLip2	pCfB5219	BB02564, BB02563	This study
pCfB7884	prDNA-Ura3d1-TPex20-HpCrtZ<-PrTefintron-TLip2	pCfB5313	BB02564, BB02563	This study
pCfB8027	prDNA-Ura3d1-TPex20-HpBKT<-PrTefintron-TLip2	pCfB5313	BB02209, BB02566	This study

Table S4. List of primers used in this study. Underlined sequences represent overhangs used for USER cloning.

Primer ID	Description	Sequence, 3´ →5´
PR-15523	PrYlFBA1 _fw (PR-15523)	CGTGCGAUAACAGTGTACGCAGTACTATAGAGG
PR-15524	PrYlFBA1 _rev (PR-15524)	ATGACAGAUTGTGTGATGTGTAGTTTAGATTTCG
PR-18214	PrTefintron_USER_rv	AGTACTGCAAAAAGUGCTG
PR-21242	SsGGPPs7_fw	ATCTGTCAU GCCACA ATGGTCGCACAAACTTTC
PR-21243	SsGGPPs7_rv	CACGCGAU TTAATGCTGACGACGTGTG
PR-18928	PrTefintron_USER <-_U1_fw	CACGCGAU AGAGACCGGGTTGG
PR-20673	PsCrtWGene2_Fw	ACTTTTTGCAGTACUAACCGCAGTCTGCCCACGCTCT GCCCAAG
PR-20675	PaCrtZGene2_Fw	ACTTTTTGCAGTACUAACCGCAGCTGTGGATCTGGA ACGCCCTGATCG
PR-20676	PaCrtZGene2_Rv	CACGCGAUTTACTTGCCAGAGGCGGGCTCGT
PR-20677	PsCrtWGene2_Rv	CACGCGAUTTAGGCGGTGTCGCCCTTAGTTCGAG
PR-21079	HpCrtZ_Ylop_forfusionPrTefintron_U2_rv	CACGCGAU TTATCGCTTAGACCAGTCC
PR-21419	HpBKT_forfusionPrTefintron_USER_EcoRI_U1_fw	ACTTTTTGCAGTACUAACCGCAG CACGTGGCCTCTGCTCTG

PR-21421	HpCrtZ_forfusionPrTefintron_USER_U2_fw	ACTTTTTGCAGTACUAACCGCAG CTGTCTAAGCTGCAGTCT
PR-21422	PrTefintron_USER_SfaAI_BamHI->_U2_fw	CGTGCGAU GCGATCGC CCGG GGATCC AGAGACCGGGTTGGCGGC
PR-21423	HpBKT_forfusionPrTefintron_USER_SfaAI_U1_rv	CGTGCGAU GCGATCGC TTAGGCCAGAGCGGGGAC
PR-7043	TJOS-25F (PR-7043)	ATCTGTCAU ATGGATTACGCGAACATCCTC
PR-7044	TJOS-25R (PR-7044)	CACGCGAU TCACAGAGGGATATCGGCTAG

Table S5. List of BioBricks used in this study. BioBricks were obtained by PCR, using the template and primers as indicated in the table.

Biobrick ID	Description	Template	Forward primer	Reverse primer	Reference
BB01559	PrYl*FBA1*->	gDNA *Y. lipolytica*	PR-15523	PR-15524	[23]
BB01569	*XdcrtE*->	p1763 (YB/I/E)	PR-7043	PR-7044	[23]
BB02093	PrTefintron_USER	gDNA *Y. lipolytica*	PR-10595	PR-18214	[23]
BB02142	*GGPPs7*	GeneString PL-6778 (SsGGPS7)	PR-21242	PR-21243	This study
BB02209	PrTefintron_USER <-	gDNA *Y. lipolytica*	PR-18928	PR-18214	[23]
BB02220	*PscrtW*	Synthetic gene string from GeneArt	PR-20673	PR-20677	[23]
BB02221	*PacrtZ*	Synthetic gene string from GeneArt	PR-20675	PR-20676	[23]
BB02563	*HpcrtZ*	Synthetic gene string from GeneArt p7642 (HpCrtZ_ylop)	PR-21421	PR-21079	This study
BB02564	Promoter for *HpcrtZ*	gDNA *Y. lipolytica*	PR-21422	PR-18214	This study
BB02566	*HpBKT*_forfusionPrTefintron_USER_SfaAI<-	GeneString p7641 (HpBKTm_ylop)	PR-21419	PR-21423	This study

Supplementary sequences

Sequences of synthetic genes used in the study.
>**GGPPs7** (geranylgeranyl pyrophosphate synthase from *Synechococcus sp.* codon-optimized for *Y. lipolytica*).

ATGGTCGCACAAACTTTCAACCTGGATACCTACTTATCCCAAAGACAACAACAAGTTGAAGAGGCC

CTAAGTGCTGCTCTTGTGCCAGCTTATCCTGAGAGAATATACGAAGCTATGAGATACTCCCTCCTGG

CAGGTGGCAAAAGATTAAGACCTATCTTATGTTTAGCTGCTTGCGAATTGGCAGGTGGTTCTGTTGA

ACAAGCCATGCCAACTGCGTGTGCACTTGAAATGATCCATACAATGTCACTAATTCATGATGACCTG

CCAGCCATGGATAACGATGATTTCAGAAGAGGAAAGCCAACTAATCACAAGGTGTTCGGGGAAGA

TATAGCCATCTTAGCGGGTGATGCGCTTTTAGCTTACGCTTTTGAACATATTGCTTCTCAAACAAGAG

GAGTACCACCTCAATTGGTGCTACAAGTTATTGCTAGAATCGGACACGCCGTTGCTGCAACAGGCCT

CGTTGGAGGCCAAGTCGTAGACCTTGAATCTGAAGGTAAAGCTATTTCCTTAGAAACATTGGAGTAT

ATTCACTCACATAAGACTGGAGCCTTGCTGGAAGCATCAGTTGTCTCAGGCGGTATTCTCGCAGGGG

CAGATGAAGAGCTTTTGGCCAGATTGTCTCATTACGCTAGAGATATAGGCTTGGCTTTTCAAATCGTC

GATGATATCCTGGATGTTACTGCTACATCTGAACAGTTGGGGAAAACCGCTGGTAAAGACCAGGCA

GCCGCAAAGGCAACTTATCCAAGTCTATTGGGTTTAGAAGCCTCTAGACAGAAAGCGGAAGAGTTG
ATTCAATCTGCTAAGGAAGCCTTAAGACCTTACGGTTCACAAGCAGAGCCACTCCTAGCGCTGGCA
GACTTCATCACACGTCGTCAGCATTAA

>**crtW** (ß-ketolase from *Paracoccus sp.* codon-optimized for *Y. lipolytica*)

ATGTCTGCCCACGCTCTGCCCAAGGCCGACCTGACCGCCACCTCTCTGATCGTGTCTGGCGGCATCA
TTGCCGCCTGGCTGGCCCTGCATGTGCACGCCCTGTGGTTTCTGGACGCCGCTGCTCACCCCATCCTG
GCTATCGCCAACTTCCTGGGCCTGACCTGGCTGTCTGTGGGCCTGTTCATTATCGCCCACGACGCCAT
GCACGGCTCTGTGGTGCCCGGACGACCCCGAGCCAACGCCGCCATGGGCCAGCTGGTGCTGTGGCT
GTACGCCGGCTTCTCTTGGCGAAAGATGATCGTGAAGCACATGGCCCACCACCGACACGCCGGCAC
CGACGACGACCCTGACTTCGACCACGGCGGACCCGTCCGATGGTACGCCCGATTCATCGGCACCTA
CTTCGGCTGGCGAGAGGGCCTGCTGCTGCCCGTGATCGTGACCGTGTACGCCCTGATCCTGGGCGAC
CGATGGATGTACGTGGTGTTCTGGCCCCTGCCTTCTATCCTGGCCTCTATCCAGCTGTTCGTGTTCGGC
ACCTGGCTGCCCCACCGACCTGGCCACGACGCTTTCCCCGACCGACACAACGCCCGATCTTCTCGAA
TCTCTGACCCCGTGTCTCTGCTGACCTGCTTCCACTTCGGCGGCTACCACCACGAGCACCATCTGCAC
CCCACCGTGCCTTGGTGGCGACTGCCCTCTACTCGAACTAAGGGCGACACCGCCTAA

>**PacrtZ** (ß-hydroxylase from *Pantoea ananatis* codon-optimized for *Y. lipolytica*)

ATGCTGTGGATCTGGAACGCCCTGATCGTGTTCGTGACCGTGATCGGCATGGAAGTGATCGCCGCTC
TGGCCCACAAGTACATCATGCACGGCTGGGGCTGGGGATGGCACCTGTCTCACCACGAGCCTCGAA
AGGGCGCCTTCGAGGTGAACGACCTGTACGCCGTGGTGTTCGCTGCCCTGTCTATCCTGCTGATCTA
CCTGGGCTCTACCGGCATGTGGCCCCTGCAGTGGATCGGAGCCGGCATGACCGCCTACGGCCTGCTG
TACTTCATGGTGCACGACGGCCTGGTCCACCAGAGATGGCCCTTCCGGTACATTCCCCGAAAGGGCT
ACCTGAAGCGACTGTACATGGCCCACCGAATGCACCACGCCGTGCGAGGCAAGGAAGGCTGCGTTT
CTTTCGGCTTCCTGTACGCACCTCCTCTGTCTAAGCTGCAGGCTACCCTGCGAGAGCGACACGGCGC
TCGAGCCGGCGCTGCCAGAGATGCCCAAGGCGGCGAGGACGAGCCCGCCTCTGGCAAGTAA

>**HpctrZ** (ß-hydroxylase from *H. pluvialis* codon-optimized for *Y. lipolytica*)

ATGCTGTCTAAGCTGCAGTCTATCTCTGTGAAGGCCCGACGAGTCGAGCTGGCCCGAGACATCACCC
GACCTAAGGTGTGCCTGCACGCCCAGAGATGTTCTCTGGTGCGACTGCGAGTGGCTGCTCCCCAGAC
CGAGGAAGCCGTGGGCACCCAGCAGGCCGCTGGCGCTGGCGACGAGCACTCTGCCGACGTGGCCC
TGCAGCAGCTGGACCGAGCCATTGCCGAGCGACGAGCCCGACGAAAGCGAGAGCAGCTGTCTTAC
CAGGCTGCCGCTATCGCCGCCTCTATCGGCGTGTCTGGAATCGCCATCTTCGCCACCTACCTGCGATT

CGCCATGCACATGACCGTCGGCGGAGCTGTGCCCTGGGGCGAAGTGGCTGGCACCCTGCTGCTGGT
GGTCGGAGGCGCCCTCGGCATGGAAATGTACGCCCGATACGCCCACAAGGCCATCTGGCACGAGTC
GCCCCTCGGCTGGCTGCTGCACAAGTCTCATCACACCCCTCGAACCGGACCTTTCGAGGCCAACGAC
CTGTTCGCCATCATCAACGGACTGCCCGCCATGCTGCTGTGTACCTTCGGCTTCTGGCTGCCCAACGT
GCTGGGCACCGCCTGCTTCGGAGCCGGCCTGGGCATCACCCTGTACGGCATGGCCTACATGTTCGTC
CACGACGGCCTGGTGCACCGACGATTCCCCACCGGACCTATCGCTGGACTGCCCTACATGAAGCGA
CTGACCGTGGCTCACCAGCTGCACCACTCTGGCAAGTACGGCGGAGCCCCTTGGGGCATGTTCCTGG
GACCTCAAGAGCTGCAGCACATCCCCGGTGCCGCCGAAGAGGTCGAGCGACTGGTGCTCGAGCTGG
ACTGGTCTAAGCGATAA

HpBKT (β-ketolase from *H. pluvialis* codon-optimized for *Y. lipolytica*)

ATGCACGTGGCCTCTGCTCTGATGGTCGAGCAGAAGGGCTCTGAGGCCGCTGCCTCTTCTCCCGACG
TGCTGCGAGCCTGGGCTACCCAGTACCACATGCCTTCCGAGTCCTCTGACGCCGCTCGACCCGCTCT
GAAGCACGCCTACAAGCCTCCAGCCTCCGACGCCAAGGGCATCACCATGGCTCTGACCATCATCGG
AACCTGGACCGCCGTGTTCCTGCACGCCATCTTCCAGATTCGACTGCCCACCTCTATGGACCAGCTG
CACTGGCTGCCCGTGTCTGAGGCCACCGCTCAGCTGCTCGGCGGATCTTCTTCTCTGCTGCACATTGC
CGCCGTCTTTATCGTGCTCGAGTTCCTGTACACCGGCCTGTTCATCACCACTCACGACGCCATGCACG
GCACCATTGCTCTGCGACACCGACAGCTGAACGACCTGCTGGGCAACATCTGCATCTCCCTGTACGC
CTGGTTCGACTACTCTATGCTGCGACGAAAGCACTGGGAGCACCACAACCACACCGGCGAGGTCGG
CAAGGACCCCGACTTCCACAAGGGCAACCCCGGACTGGTGCCCTGGTTCGCCTCTTTCATGTCCTCT
TACATGTCTCTGTGGCAGTTCGCCCGACTGGCCTGGTGGGCCGTCGTCATGCAGATGCTGGGCGCTC
CCATGGCCAACCTGCTGGTGTTCATGGCCGCTGCTCCCATCCTGTCCGCCTTCCGACTGTTCTACTTC
GGCACCTACCTGCCTCACAAGCCCGAGCCTGGACCTGCCGCCGGATCTCAGGACATGGCCTGGTTC
CGAGCCAAGACCTCTGAGGCTTCTGACGTGATGTCTTTCCTGACCTGCTACCACTTCGACCTGCATTG
GGAGCATCATCGATGGCCCTACGCTCCCTGGTGGCAGCTCCCTCACTGCCGACGACTGTCTGGCCGA
GGACTGGTCCCCGCTCTGGCCTAA
